# Arabic Translation and Rasch Validation of PROMIS Anxiety Short Form among General Population in Saudi Arabia

**DOI:** 10.3390/bs14100916

**Published:** 2024-10-09

**Authors:** Hadeel R. Bakhsh, Monira I. Aldhahi, Nouf S. Aldajani, Tahera Sultana Davalji Kanjiker, Bodor H. Bin Sheeha, Rehab Alhasani

**Affiliations:** 1 Department of Rehabilitation Sciences, College of Health and Rehabilitation Sciences, Princess Nourah bint Abdulrahman University (PNU), P.O. Box 84428, Riyadh 11671, Saudi Arabia; hrbakhsh@pnu.edu.sa (H.R.B.); mialdhahi@pnu.edu.sa (M.I.A.); 438001243@pnu.edu.sa (N.S.A.); rsalhasani@pnu.edu.sa (R.A.); 2 School of Public Health, Boston University, Boston, MA 02118, USA; taheradk@bu.edu

**Keywords:** Arabic, anxiety, outcome measures, mental health, PROMIS, Rasch analysis, psychometrics, cultural adaptation, translation

## Abstract

Background: This study aimed to translate, culturally adapt, and psychometrically validate the PROMIS Anxiety Short Form 8a item bank into Arabic for the general population of Saudi Arabia. Methods: The PROMIS Anxiety Short Form was translated according to the FACIT group method. Second, psychometric validation was conducted on a convenience sample of 322 participants (mean age, 26 ± 10.4 years; predominantly female) from the general population in Saudi Arabia. Rasch analysis (Winsteps^®^ version 5.6.1) was used to examine category functioning, item fit, the person separation index, item difficulty, unidimensionality, and local dependency. Results: Translation and cultural adaptation demonstrated that most of the items were culturally suitable and conveyed the same underlying concepts as the original scale. The five response categories of the scale satisfied the category functioning criteria, and all items fit the underlying construct, with the exception of one item that demonstrated a misfit. The item difficulty demonstrated poor targeting for the sample population; however, the person separation index and reliability were good (2.67 and 0.88, respectively) and no local dependency was noted. Conclusions: The Arabic translation of PROMIS-A SF8a demonstrated good structural validity and psychometrics, making it a valuable tool for screening anxiety in Arabic-speaking populations. The application of this outcome measure shows promise for healthcare professionals and patients alike, as it contributes to the provision of high-quality care and formulation of appropriate treatment plans.

## 1. Introduction

The global prevalence of mental disorders is increasing, making them the second most common cause of disability among individuals and posing a significant public health challenge [1]. The Saudi National Mental Health Survey indicates that mental health disorders are an increasing issue in Saudi Arabia, with approximately 34% of the population having experienced a mental disorder at some point in their lives [2,3]. Moreover, the same survey found that mental health conditions are more prevalent among young adults (aged between 15 and 34 years), with 40% satisfying the criteria for a mental disorder, which is nearly twice as high as the older adult population (50–65 years) [2,3]. The World Health Organisation’s International Classification of Disease defines anxiety as a disorder characterised by an intense and excessive focus on an ‘anticipated threat’. The symptoms include apprehension, motor tension, and autonomic overactivity [4]. Reports indicate that the risk of anxiety is high in Saudi Arabia, coupled with low diagnosis rates, underscoring the need for enhanced mental health promotion, early detection, and improved accessibility to treatment [2].

Previous studies have highlighted gender disparities in anxiety and revealed that compared to males, Saudi women have a significantly higher risk of developing anxiety disorders, including separation anxiety disorder, obsessive–compulsive disorder, and anxiety disorder [2]. A recent study on mental health screening prevalence in Saudi Arabia found a low prevalence of diagnosed and treated anxiety, with only 0.5% of participants currently diagnosed and receiving treatment for depression and anxiety [2]. Consequently, highlighting the need for anxiety screening tools, especially with the growing focus on mental health research in the Arab region, requires the development of culturally relevant and psychometrically sound tools for accurate diagnoses and treatments.

Patient-reported outcome measures (PROMs) are often used as screening and assessment tools for anxiety and can provide critical information on patients’ perceived quality of life, symptomology, and overall well-being [5]. Among the most commonly used PROMs (alongside clinical assessments) are the Hospital Anxiety and Depression Scale (HADS) [6], Generalised Anxiety Disorder 7 (GAD-7) scale, and Depression Anxiety Stress Scale (DASS) scale [4,7,8]. Although these measures have been psychometrically tested and studied extensively, they have some limitations. For instance, most measures focus on symptom severity rather than on a holistic view of the patient’s functioning and quality of life. Moreover, the HADS combines anxiety and depression, which can complicate the interpretation of anxiety-specific results. The GAD-7 scale has been found to have low specificity among Arabic-speaking Lebanese psychiatric outpatient samples. Furthermore, the DASS-21 scale has demonstrated poor discriminant validity across different Arab populations [9,10]. Thus, current anxiety measurement tools suffer from various limitations, including lengthy administration times, low specificity, and limited discriminant validity [9,10,11,12,13]. Therefore, the severity and impact of anxiety on daily activities should be measured using instruments with sufficient psychometric properties and a low completion burden for patients [14]. Furthermore, the measurement of anxiety should be standardised in research and clinical practice to enable a comparison of the burden of disease and treatment within and across populations [14].

Consequently, to improve the quality of mental health measurements and standardise their measurement across populations, the Patient-Reported Outcomes Measurement Information System (PROMIS), an initiative launched by the American National Institute of Health, is becoming a widely recognised set of assessment tools used in healthcare research and clinical practice [15,16]. PROMIS aims to develop valid and reliable PROMs and item banks for mental health disorders, physical functions, and social participation [17]. The PROMIS Anxiety (PROMIS-A) item bank was built on items from existing PROMs that had undergone rigorous testing identified in an extensive search of the literature, as well as in focus groups with a mixed sample of patients [11,12,13,18,19,20,21,22,23,24]. In a general population sample, the Dutch–Flemish PROMIS item banks for depression and anxiety were compared with the Brief Symptoms Inventory to evaluate their effectiveness in assessing anxiety [20,22,23]. The results showed that PROMIS-A had a greater ability to detect actual changes than the Brief Symptoms Inventory scale, highlighting its utility in assessing and monitoring anxiety across different populations [20]. Moreover, the PROMIS-A was found to be effective in detecting and assessing anxiety in cancer patients and in enhancing patient care by identifying and addressing anxiety symptoms that can negatively impact treatment adherence and quality of life [11].

To date, the tool has been translated into Brazilian Portuguese [19], German [25], and Dutch– Flemish [20], and adaptations to other languages are underway. Although some existing scales have been validated, there is still a demand for cross-cultural validation to ensure their applicability to Arabic populations, particularly across different populations [26]. However, all of these studies addressing the development and psychometric properties of the PROMIS-A item bank in multiple populations were performed in the US and Europe. There is no evidence yet for the psychometric properties of the Arabic PROMIS-A measure outside the US and Europe. There is also limited evidence of measurement invariance across demographic variables and across countries (cross-cultural validity). This is important because item parameters may vary across countries, which could impact scores and hinder comparisons between groups that differ with respect to demographic variables or cultural backgrounds.

Therefore, this study aimed to translate and psychometrically validate the Arabic version of the PROMIS Anxiety Short Form (PROMIS-A SF) for the general population of Saudi Arabia. It aims to facilitate large-scale international implementation of this item bank as a short form in research and clinical practice. The lack of culturally appropriate and robust PROMs and of standardisation of such measures among Arabic populations continues to raise concerns about the diagnostic accuracy of available screening instruments and hinders comparative research in Arabic-speaking countries [26,27]. This study could improve the detection rates of anxiety disorders, facilitate effective treatment planning and evaluation, enhance communication with patients, and advance evidence-based care and research on mental health in Saudi Arabia.

## 2. Materials and Methods

### 2.1. Study Design

This cross-sectional, methodological study consisted of two distinct phases. The first phase involved a translation process and pilot testing (comprehensibility), following the specifications outlined by the PROMIS Health Organization. Consequently, the translation was conducted by contracting with Functional Assessment of Chronic Illness Therapy Translation (FACITtrans), which also follows the International Standard Organization (ISO) 17100 for professional competencies and translation qualifications of linguists [28]. The second phase involved psychometric validation, which was conducted in accordance with the guidelines outlined by the Rasch Reporting Guideline for Rehabilitation Research (RULER) [29,30].

### 2.2. Ethical Consideration and Licensing

The PROMIS Health Organisation (PHO) permission to translate the PROMIS Emotional Distress–Anxiety Item Bank into Arabic was obtained in April 2022. Princess Nourah Bint Abdulrahman University (PNU) (KACST, KSA: HAP-01-R-059) and King Abdullah Bin Abdulaziz University Hospital (KAAUH) (RO-2023-P-019) Institutional Review Boards approved this study. This study was conducted in accordance with the principles of the Declaration of Helsinki. The Arabic questionnaire can be requested from www.healthmeasures.net (accessed on 1 August 2024).

### 2.3. Measures

Participants completed a questionnaire consisting of three sections. The first section was concerned with sociodemographic data such as age, sex, height, weight, education level, employment status, and marital status. The second section concerned their health profiles and included questions about non-communicable diseases (hypertension, diabetes, musculoskeletal diseases, etc.).

The third section was PROMIS Emotional Distress–Anxiety (PROMIS-A). The PROMIS-A item bank was designed to assess anxiety-related emotional distress. It consists of 29 items that were developed and calibrated using item response theory [17]. These items were written with a seven-day timeframe and five response options, reflecting the frequency from 1 = never to 5 = always. The PROMIS-A items focus on fear, anxious misery, hyperarousal, and somatic symptoms related to arousal [17]. The measure is also available in short-form versions, involving eight, six, or four items to reduce the response burden. For the purpose of this study, short form 8a (SF8a) was used [17]. A T-score of 50 corresponds to the mean score of the overall US population, with a standard deviation of 10. Higher scores correspond to elevated levels of anxiety [17]. According to PROMIS standards, anxiety levels between 55 and 60 were categorised as mild problems, scores between 60 and 70 were considered moderate problems, and scores above 70 were classified as severe problems.

#### 2.3.1. Translation Process

Linguists, translators, proofreaders, and cognitive interviewers from FACITtrans collaborated on Arabic translations (Supplementary Material S1). The FACIT translation involved two native Arabic-speaking professional translators (T1 and T2) who independently translated source items from English to Arabic. Second, a third independent translator (T3) harmonised the two forward translations to create version 1.0. Third, a native English-speaking translator (T4) conversant in the target language back-translated reconciled version 1. The translator had no knowledge of the item definitions or original form. The translation project manager thoroughly compared the original and back-translated English versions to identify differences. The Arabic language coordinator oversaw the final translation by analysing the previous phases and FACITtrans team feedback. Finally, the FACITtrans team and PROMIS Statistical Centre checked the translated version for comparability and consistency with prior translations and items.

#### 2.3.2. Pilot Study (Comprehensibility)

The pilot test included 30 native Arabic speakers from Saudi Arabia, Morocco, Kuwait, Jordan, and Egypt with equal participation from each nation. The study participants were native Arabic speakers, aged 18 years or older, who were able to provide verbal consent. R.A., M.I.A., H.R.B., and B.H.B. interviewed them. The participants completed the questionnaire independently and were interviewed using scripts. Interviewees wrote a cognitive debriefing report on their thoughts and ideas. After receiving the revision comments, the translation project manager and language coordinator made the final recommendations for changes or translational solutions. The translation project manager provided a report on the comprehensibility of the translated items for PROMIS statistical quality approval.

### 2.4. Phase II Validation and Psychometric Testing

#### 2.4.1. Participants and Procedure

This multicentre study was conducted using a convenience sample of the general population attending Princess Nourah Bint Abdulrahman University (PNU) and King Abdullah Bin Abdulaziz University Hospital (KAAUH) from April to June 2023. Individuals were invited to participate if they were (1) older than 18 years of age and (2) able to read and comprehend Arabic. The presence of cognitive impairments that would interfere with the completion of the questionnaire was considered an exclusion criterion.

A total of 350 participants were invited to participate in this study; three participants were excluded because they were under 18 years old, and 25 declined to participate. The final analysis included 322 participants enrolled in the study.

The research team interacted with the community members in the reception areas of the rehabilitation departments of KAAUH, college lobbies, and libraries at PNU. Once the participants consented to the study, they were asked to complete a computerised questionnaire using Microsoft Forms, which took approximately five to seven minutes to complete.

#### 2.4.2. Data Analysis

Descriptive statistics, including frequency, percentage, mean, and standard deviation (SD), were used to provide an overview of the demographic characteristics of the sample. Rasch analysis was performed using Winsteps version 5.6.1 [31], and the rating scale model was applied, as the log–likelihood ratio test for model comparison revealed no advantage of employing the sophisticated partial credit model. A sample size of 350 participants allowed for the stable calibration of items within a logit of ±0.5, with 99% confidence [32], and allowed us to investigate the following aspects of the scale.

**Figure 1:**
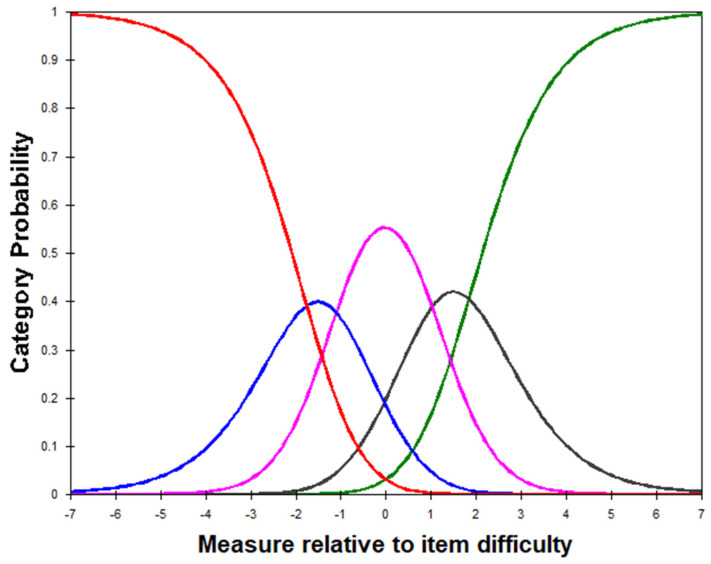
Probability curves for the five response categories of PROMIS-A short-form 8a. Note: Red: Never; Blue: Rarely; Pink: Sometimes; Black: Often; Green: Always.

Rating scale category (threshold) ordering—An analysis was conducted based on established criteria proposed by Linacre (1999) and Wolfe and Smith (2007) [33,34]. The primary focus was to investigate the ordered response thresholds for each category, which represented the transition points between the adjacent categories. It is important to ensure that the category response increases with the corresponding transition point (threshold) between categories (Figure 1), which reflects an increase in the underlying trait (in this case, greater anxiety).

Item fit, targeting, and item difficulty—Chi-square fit statistics were used to assess the alignment of individual items using the Rasch model. Acceptable fit was defined based on sample size, with mean square values ranging from 0.8 to 1.2 and standardised z-values (ZStd) within ±2.0 indicating good fit. Items exhibiting misfit were identified when both the MnSq and ZStd values fell outside the specified ranges [34]. Underfitting (higher values) items indicate unpredictable high variability, whereas overfitting (lower values) items indicate redundancy or predictable patterns [35].

**Figure 2:**
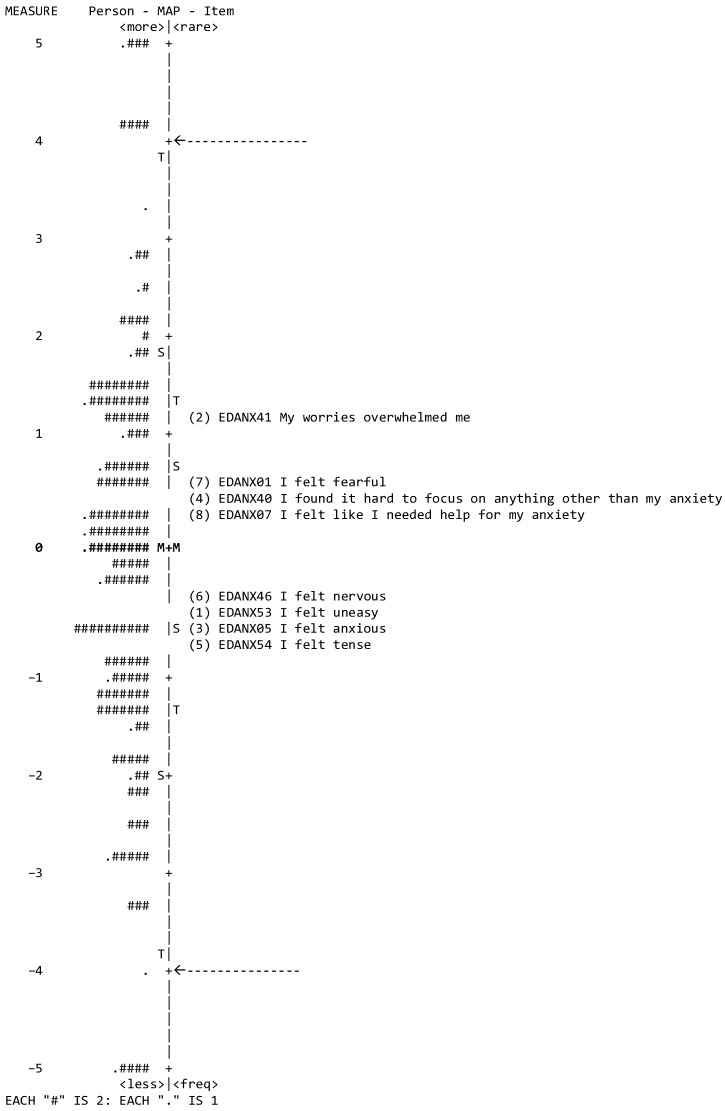
Wright’s map participant ability and item difficulty map of the PROMIS-A-SF.

Item difficulty refers to how ‘hard’ or ‘easy’ it is for a participant to agree with or endorse an item in a survey or test. In the context of questionnaires, it indicates the level of the underlying trait (in this case, anxiety) that a participant needs to have to answer an item in a certain way. Participants’ abilities were estimated in logit units to quantify the difficulty associated with each item and the location of individual participants on a common interval scale, as illustrated in Wright’s map (Figure 2). A higher measure estimate indicates a higher ability (i.e., anxiety) and more difficult items. Items are considered ‘on target’ with the sample when the person mean is within 0.5 logits of the item mean [36].

Reliability and separation indices: the person separation index (PSI) and reliability indicate the capacity to discriminate between three or more levels of ability (i.e., anxiety), which are marked by PSI 2.0 or higher [37]. The following formula was used to determine the number of strata: strata = [4 × (person separation index) + 1]/3 [38].

Principal component analysis of residuals (PCAr): to examine whether other factors significantly contributed to the residuals beyond the primary Rasch factor, PCAr was conducted. The presence of additional factors was indicated if the Rasch factor accounted for 50% of the variation and if an eigenvalue of the first contrast was greater than 2. Local dependency between items was flagged when the correlation value was >0.30 [39].

## 3. Results

### 3.1. Translation Process and Pilot Study (Comprehensibility)

The participants in the pilot testing for comprehensibility had an average age of 35.2 years (SD ± 10.5 years), with equal numbers of male and female participants. Most items were culturally suitable and conveyed the same underlying concepts as those of the original English scale. Three items were identified as unclear for all participants, warranting further examination (Supplementary Material S2). The item ‘It scared me when I felt nervous’ posed clarity issues for three participants, while the item ‘Many situations made me worry’ had one concern related to word order, and two participants encountered difficulties with the item ‘I was easily startled’, who found the term ‘puzzled/astonished’ unclear and rephrased it with ‘afraid’. In light of these findings, all recommendations for rewording underwent thorough deliberation and discussion between the researchers and language consultant until a consensus was reached. The final, linguistically validated Arabic questionnaires are accessible for reference at http://www.healthmeasures.net (accessed on 1 August 2024).

### 3.2. Phase II Validation and Psychometric Testing

Demographic characteristics of the participants are presented in Table 1. A total of 322 participants were included in the analysis, with an average age of 26 ± 10.4 years. Most participants were female, accounting for 89% of the sample. Three-quarters of the participants were single (75%), and the majority were identified as students (66%); hence, most of the participants were undergraduates (62.1%), and most were from Riyadh (80.4%). In the general population, the PROMIS-A SF8a Arabic version had an average T-score of 62 ± 9.2.

Rating scale functioning: the five response options of the PROMIS-A SF8a satisfied the category functioning criteria defined by Linacre (Table 2 and Figure 1). The probability curves of the subcategories demonstrate the distinction between each category along the attribute measurement from one to five.

Item fit and item difficulty: the structural validity of the PROMIS-A SF8a scale was supported, as indicated by the acceptable fit of most items to the Rasch model (Table 3), with the exception of one item which was flagged for misfitting values: item 5, EDANX05, ‘I felt anxious’ (infit MnSq 0.62). The easiest item was #5, EDANX05, ‘I felt anxious’, and the most difficult item was #2, EDANX41, ‘My worries overwhelmed me’. Item difficulty estimates spanned 1.79 logits (from −0.65 to 1.14), whereas person ability (degree of anxiety) spanned 8 logits (from −4.0 to 4.0 non-extremes), with a mean person ability of −0.04 below the mean item difficulty (anchored at 0), indicating poor person-to-item targeting. Additionally, a Rasch nomogram was constructed based on item calibrations to transform the raw scores of the PROMIS-A SF8a into a linear estimation of anxiety, as demonstrated in Wright’s map (3).

**Separation indices**: PSI and person reliability were 2.67 and 0.88, respectively, which indicates that the PROMIS-A SF8a scale items can separate individuals into at least three statistically distinct levels (strata) within our sample (number of strata = [4 × separation index + 1]/3 = 3.89). Item separation and reliability were 8.08 and 0.98, respectively, with an excellent Cronbach’s α of 0.92. Seven participants (2.2%) obtained the maximum score, and nine participants (2.8%) obtained the minimum score. Thus, floor and ceiling effects were insignificant.

**Principal component analysis of the residuals (PCAr)** revealed that the variance attributable to the Rasch measure accounted for 62.5% of the total variance, with an associated eigenvalue of 13.31 and a residual variance in the first contrast of 7.3% (eigenvalue = 1.5), indicating the presence of one dominant underlying factor.

The vertical line represents the variable measure in linear logit units. Item difficulty is distributed in the right-hand column (the difficulty estimate for each item represents the mean calibration of the threshold parameters according to the rating scale model), and the personal measures are distributed in the left-hand column, which indicates the individual’s ability (i.e., anxiety) along the variable. From the bottom, measures indicate ‘less anxiety’ for participants and ‘easier’ items to be endorsed, whereas ‘higher anxiety’ and ‘more difficult’ items to be endorsed are located at the top. Each ‘#’ denotes two persons, whereas ‘.’ denotes one person. The average difficulty of the test items was anchored at 0 logits (indicated by M). Accordingly, a participant with an average ability is indicated by ‘M′. The two arrows represent the threshold boundaries for item difficulty (non-extreme).

## 4. Discussion

The objective of this study was to translate the PROMIS-A SF8a into Arabic and to validate the scale in the general population using Rasch analysis. In the translation and pilot testing phases, attention was paid to three specific items within the PROMIS-A SF8a which required further revision. Translational challenges were encountered with items EDANX03 (‘It scared me when I felt nervous’) and EDANX48 (‘Many situations made me worry’) because of differences in word order between Arabic and English. For instance, in the case of item EDANX03, the phrase ‘When I get nervous, I feel scared’ proved challenging to convey clearly in Arabic when rendered in the present tense. Therefore, to enhance comprehensibility and ensure semantic alignment, the translation process judiciously employed a shift to the past tense.

Moreover, the primary concern identified in item EDANX20 (‘I was easily startled’) pertains to utilising a keyword characterised by multiple common interpretations. Consequently, participants’ feedback underscored the necessity of an alternative keyword with a more precise and contextually congruent meaning. The word ‘startled’ in English generally means being surprised or shocked suddenly, often due to something unexpected. However, translating this exact meaning into Arabic can depend on the context.

For instance, the word ‘مندهش’ (Mundahish): translates to ‘astonished’ or ‘amazed,’ implying surprise or wonder but lacking the suddenness or jarring quality of ‘startled,’ whereas the word ‘مرتبك’ (Murtabik) translates to ‘puzzled’ or ‘confused,’ which may convey an element of surprise but not necessarily the shock or abruptness of being startled. Therefore, in the case of item EDANX20 (‘I was easily startled’), it was difficult to translate ‘puzzled/astonished’ into Arabic, primarily because the equivalent terms are predominantly associated with conveying a sense of confusion or perplexity rather than evoking the sensation of fear or being frightened. In contrast, the term ‘startled’ in Arabic embodies an unequivocal and explicit connotation, specifically denoting a state of fear or experiencing fright.

Rasch analysis validation of the PROMIS-A SF8a confirmed that the five response category thresholds were ordered according to the criteria described by Linacre [34] and demonstrated sufficient discrimination between each category, indicating that they effectively differentiated between varying levels of anxiety. Our findings align with the literature demonstrating the strong discriminative ability of PROMIS scales [15,40]. Pilkonis et al. [17] confirmed the appropriate ordering of response categories in a larger item pool, which informed the short form used in our study. Thus, the consistent performance of the response categories across studies underscores the robustness of the PROMIS anxiety items in capturing various levels of anxiety. Additionally, the probability curves of the response categories provided further evidence of clear distinctions in the attribute measurements of anxiety. These curves visually depicted the probability of endorsing each category at different levels of anxiety, showing a systematic and distinguishable progression from one category to another. This finding reinforces the notion that the rating scale structure of the PROMIS-A SF8a scale satisfies category functioning criteria.

The fit of the items to the Rasch model in our study was generally acceptable, with six out of eight items demonstrating appropriate fit, thus providing support for the structural validity of the PROMIS-A SF8a scale. Item #5, EDANX05, ‘I felt anxious’, demonstrated a misfit and was specifically flagged for overfit, while item #6, EDANX46, ‘I felt nervous’, was flagged for underfit. This discrepancy implies that the item may not fully capture the intended construct of anxiety as effectively as other items on the scale. Our findings are consistent with those of Bebber et al. [41], who utilised item response theory through a graded response model, analysing a comprehensive item bank, and reported that only 9 out of 29 items fit adequately within the Dutch general sample, and 1 out of 29 anxiety items fit within the Dutch clinical sample, suggesting that certain items may not function equivalently across cultural contexts. However, none of these items were similar to those that were misfitted in our study. Additionally, Flens et al. [42] reported a sufficient item fit using the graded response model for the PROMIS Anxiety item bank. However, this contrasts with Liuzza et al. [43] and Pilkonis et al. [44], who found no significant item misfit in their samples using the four-item short form and the item bank, respectively.

Wright’s map, which transforms the raw scores into a linear estimation of anxiety, provides graphical representation that aids in better understanding and interpretation of the spread of item difficulty and participants’ abilities (i.e., anxiety) on the PROMIS-A SF8a scale. In this study, the map showed relatively poor item targeting (item estimation, 1.79 logits) for a wide range of a person’s abilities (ability estimation, eight logits), indicating that items are unable to capture various levels of anxiety presented in this sample, perhaps due to the limited number of items measuring anxiety (8). Items related to more general and common anxiety symptoms, such as ‘feeling nervous’, tended to have lower difficulty parameters, meaning they were endorsed more easily by respondents with even mild anxiety. By contrast, items that reflect more severe anxiety symptoms, such as ‘feeling panic’, tend to have higher difficulty parameters, indicating that they are endorsed only by respondents with higher levels of anxiety. Unfortunately, due to the limited number of studies evaluating the psychometrics of PROMIS-A and due to it being conducted in different contexts and populations, a direct comparison is not possible at this stage.

The reliability analysis of the PROMIS-A SF8a scale revealed excellent reliability. The person and item separation indices (2.67 and 8.08, respectively) indicate that the scale can differentiate at least three levels of anxiety (mild, moderate, and severe). In addition, the results confirmed excellent internal consistency (Cronbach’s α = 0.92), consistent with those reported in other studies (Cronbach’s α = 0.89–0.97) [15,23,40,43,45]. Therefore, the results of this study indicate high consistency and stability of the scale measurements.

The results of PCAr confirmed the unidimensionality of PROMIS-A, which is consistent with the reported literature using confirmatory and exploratory factory analysis, with CFI ranging between 0.95 and 0.98, RMSEA values typically between 0.03 and 0.05, and SRMR values below 0.08, confirming excellent model fit across various populations [19].

Additionally, our study provides Saudi reference values for the PROMIS-A measure. A T-score of 61 represents the average score of the Saudi general population, which is quite high compared to the average T-score of the US (50 ± 10), Dutch (49.9 ± 10.1), and Italian (48.33 ± 8.22) populations [17,46]. This discrepancy suggests that the Saudi population may report higher levels of anxiety, or that cultural differences influence the perception and reporting of anxiety symptoms. In the context of determining appropriate cutoff scores for clinical use, Recklitis et al. [47] offered valuable insight and validated the PROMIS-A SF8a against a structured clinical diagnostic interview in young adult cancer survivors; they found that a T-score of 53.2 achieved a sensitivity of 88% but only a specificity of 65%. This cut-off was identified as the closest to meeting the study’s criteria for a screening measure, where high sensitivity (≥85%) and moderate specificity (≥75%) are typically desired to minimise missed cases of anxiety. Moreover, the study highlights the trade-offs of using different cut-off points for anxiety screening. Higher cutoffs, such as ≥64.5, increase specificity but reduce sensitivity, which is better for minimising false positives. Using a T-score of 61 as a cutoff in the Saudi population could lead to more false negatives, whereas lower cutoff scores might increase false positives and unnecessary referrals. Thus, it is crucial to choose cutoff points that balance sensitivity and specificity, considering cultural differences. Future research should assess the suitability of these cut-off scores for the Saudi population and explore the need for culturally adapted screening tools.

Overall, the PROMIS scales have several advantages over current legacy scales for anxiety. First, PROMIS scales are applicable across the general population and various clinical groups, as well as for individuals with multimorbidity, rare diseases, or without a definite diagnosis [15,16,23]. Consequently, this enables the comparison of clinical groups, benchmarking, and improvement of quality of care. Second, PROMIS-A item banks can be used as a computerised administered test (CAT), which reduces the response burden while maintaining high measurement precision and, as such, is valuable in clinical practice [47,48]

### Strengths and Limitation

This study is the first to translate and validate the PROMIS-A SF8a into Arabic, thereby expanding its applicability to Arabic-speaking populations and contributing to broader international use of PROMIS measures. Moreover, the study’s findings on the psychometrics of PROMIS-A SF8a offer valuable insights into the clinical utility of this tool. This directly affects clinical practice by informing the development of more accurate screening protocols in diverse populations.

The findings of this study are preliminary owing to the limited research on Arabic PROMIS-A; therefore, this study has several limitations. First, the analysis was conducted using a convenience sample from one country (Saudi Arabia) and a sample that was predominantly female, which could have influenced the interpretation of the findings and limited the generalisability of the validation results to other contexts.

Future studies should aim to recruit a more representative sample, with a balanced proportion of male and female participants. This can be achieved through targeted recruitment strategies, such as using different recruitment centres or considering oversampling or stratified sampling techniques, to ensure an adequate representation of both sexes in the study sample. Furthermore, this study excluded participants with psychiatric mood and anxiety symptoms. Further studies should include participants with diverse degrees of emotion to more accurately extrapolate the findings, examine measurement invariance, and target items to participants’ levels of anxiety more closely. Moreover, involvement of a psychiatrist is imperative during the recruitment process to assess and confirm the diagnosis of mood and anxiety disorders. Additionally, it is necessary to gather evidence regarding the psychometric features of the PROMIS-A measure in other countries to facilitate the comparison of healthcare outcomes between different countries. Therefore, it is highly desirable to replicate this study in different cultural groups using a representative sample.

## 5. Conclusions

Arabic translation and validation of the PROMIS-A SF8a scale is a crucial step in making it accessible to Arabic-speaking communities. With an emphasis on cultural adaptation to the general Saudi population, the study highlighted the validity, reliability, and general applicability of the scale for assessing anxiety. While the scale performed well overall, future studies should focus on targeting item difficulty and evaluating the scale in different populations and contexts to ensure an accurate and standardised anxiety assessment and screening.

## Figures and Tables

**Table 1:** Sociodemographic characteristics of the study sample (n = 322).

Variables	n (%)
T-scores, mean (±SD)	62 (±9.2)
Age (years), mean (±SD)	26 (±10.4)
Sex: female (%)	286 (89%)
**Marital Status**	
Single	241 (75%)
Married	74 (23%)
Divorced	4 (1%)
Widowed	2 (1%)
**Occupation**	
Unemployed	38 (12%)
Student	213 (66%)
Military	4 (1%)
Public sector	27 (8%)
Private	18 (6%)
Other	49 (15%)
**Education Level**	
High school	95 (29.5)
Undergraduate	200 (62.1)
Graduate/postgraduate	27 (8.4%)

**Table 2:** PROMIS-A-SF summary of rating scale diagnostics.

Category Label	Measure	Andrich Threshold	Infit MnSq	Outfit MnSq	Observed Count (%)
1 Never	−3.13	None	1.00	1.03	447 (17%)
2 Rarely	−1.51	−1.76	1.01	0.95	452 (18%)
3 Sometimes	−0.02	−1.10	0.94	0.90	814 (32%)
4 Often	1.50	1.02	1.04	1.03	466 (18%)
5 Always	3.18	1.84	1.01	1.01	397 (15%)

**Table 3:** PROMIS-A-SF item calibration.

Items	Measure (SE)	Infit	Outfit
		MnSq (ZSTD)	MnSq (ZSTD)
(6) EDANX46, I felt nervous	−0.54 (0.07)	1.30 (3.52)	1.29 (3.08)
(4) EDANX07, I felt like I needed help for my anxiety	0.32 (0.07)	1.11 (1.38)	1.06 (0.70)
(8) EDANX40, I found it hard to focus on anything other than my anxiety	0.43 (0.07)	1.10 (1.29)	1.09 (1.03)
(7) EDANX01, I felt fearful	0.46 (0.07)	1.05 (0.63)	1.02 (0.21)
*(2)* EDANX41, *My worries overwhelmed me*	*1.14 (0.08)*	*0.99 (* *−* *0.08)*	*0.94 (* *−* *0.62)*
(1) EDANX53, I felt uneasy	−0.52 (0.07)	0.89 (−1.40)	0.97 (−0.37)
(3) EDANX54, I felt tense	−0.63 (0.07)	0.90 (−1.31)	0.88 (−1.37)
***(5) EDANX05, I felt anxious***	***−*** ***0.65 (0.07)***	***0.62 (*** ***−*** ***5.58)***	***0.61 (*** ***−*** ***5.05)***

Acceptable fit range defined as MnSq 0.7–1.3. Measure is the calibration of item difficulty. SE: standard errors. Positive values indicate more difficult items and that it is less likely it is for participants to obtain a high score (i.e., to demonstrate a high anxiety level). Bold = overfitting or underfitting. Italic = easiest and most difficult.

## Data Availability

The data presented in this study are available upon request from the corresponding author. The data are not publicly available due to restrictions; e.g., the data many contain information that could compromise the privacy of research participants.

## Supplementary Materials

The following supporting information can be downloaded at https://www.mdpi.com/article/10.3390/bs14100916/s1: Figure S1: FACIT Translation Process; Table S1: The FACITtrans Arabic translation team; Table S2: Item Adjustments in the Item Bank Translation Process.
