# Supplementary material for: Arabic Translation and Rasch Validation of PROMIS Anxiety Short Form among General Population in Saudi Arabia
Source: Behav Sci (Basel). 2024 Oct 9;14(10):916. doi: 10.3390/bs14100916 (PMC11505420; doi:10.3390/bs14100916)
Supplement: Supplementary file 1 [file behavsci-14-00916-s001.zip › behavsci-3209742-supplementary s1.pdf]

Figure S1 FACIT Translation Process

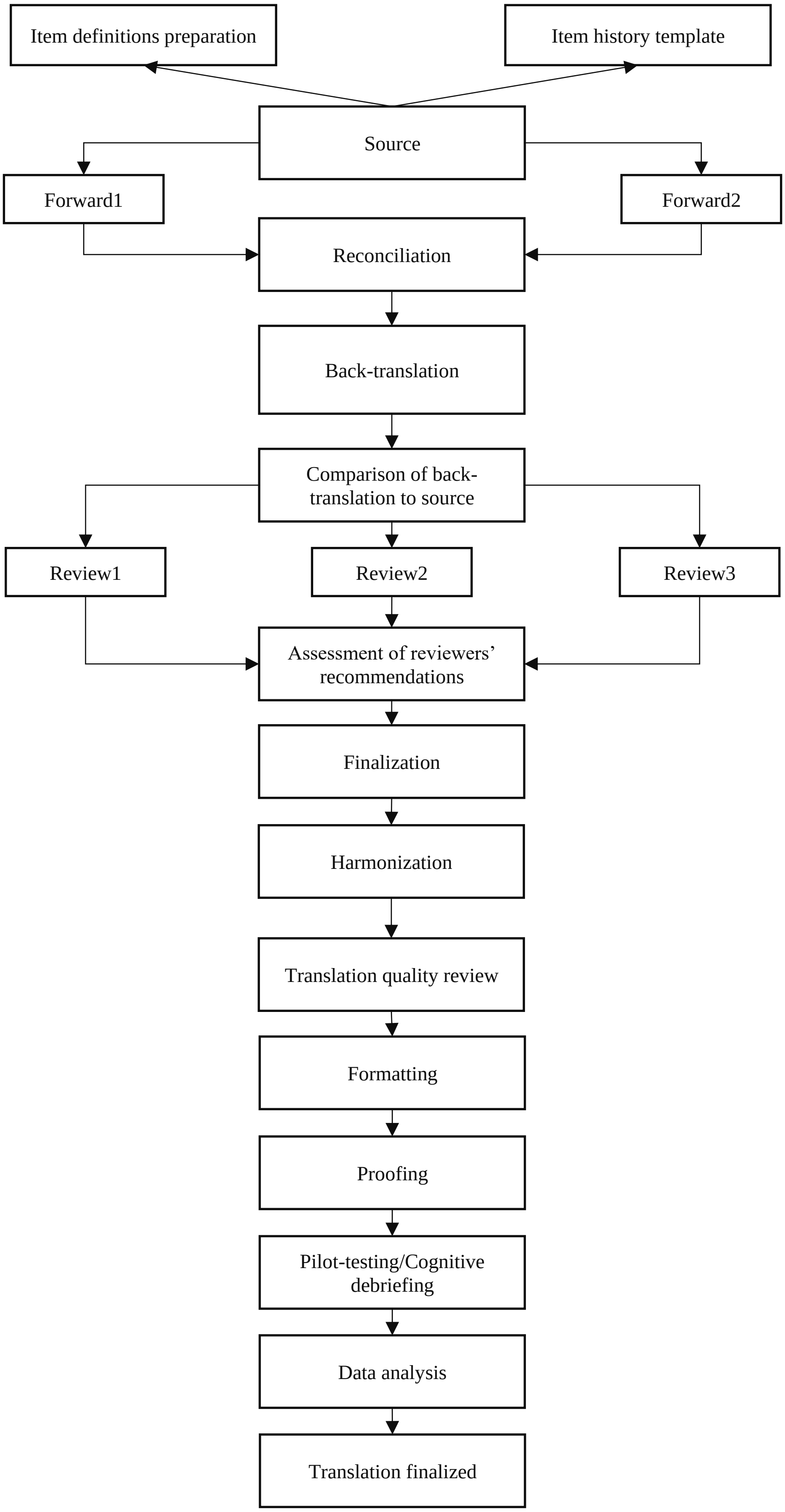

Table S1 The FACITtrans Arabic translation team

**Role***	**Qualification**	**Title & Profession**
Translation Account Manager FACITtrans	MBA	Director, PROMIS Lead
Translation Project Coordinator FACITtrans	BA	Senior COA Translations Manager - Life Sciences, PROMIS Specialist
Translation Project Manager FACITtrans	BA, Spanish Linguistics	Senior COA Translation Project Manager – Life Sciences
Forward 1	BA, Languages and Translation, Simultaneous Interpretation (English < >Arabic)	Senior Translator, Copywriter & Proofreader Professional and Translator Interpreter
Forward 2	MA, Linguistics	Professional Translator and Interpreter
Reconciler/Proofreader	Ph.D., Linguistics	Professional Linguist and Translator
Back Translator	MA, Diplomacy<br>BA, Medical Technology	Professional Translator<br>16 years full immersion in Arabic public school system and 3.5 years of undergraduate education (nursing) at King Abdul Aziz University, Jeddah Saudi Arabia
Reviewer 1	Ph.D., Linguistics	Linguist and researcher<br>Pragmatics, sociolinguistics, discourse analysis, ideology, identity, and translation studies
Reviewer 2	Ph.D., Linguistics	Professional Linguist and Translator
Reviewer 3, Language Coordinator, Proofreader 1	DDS<br>MA, Biblical Studies	Professional Translator and Interpreter<br>Close to 30 years' experience specializing in medical, legal and religious translation
