# Supplementary material for: Arabic Translation and Rasch Validation of PROMIS Anxiety Short Form among General Population in Saudi Arabia
Source: Behav Sci (Basel). 2024 Oct 9;14(10):916. doi: 10.3390/bs14100916 (PMC11505420; doi:10.3390/bs14100916)
Supplement: Supplementary file 1 [file behavsci-14-00916-s001.zip › behavsci-3209742-supplementary s2.pdf]

Table S2 Item Adjustments in the Item Bank Translation Process

Source item	English equivalent	Translation issues/ Cognitive debriefing feedback	Reasons for adaptation
PROMIS Anxiety Item Bank	**EDANX03 It scared me when I felt nervous**	ARA638<br>The sentence is not clear because it is not justified that I get scared when I get nervous.<br><br>ARA639<br>When I got nervous, I got scared (unclear meaning of the sentence)<br><br>ARA640<br>It means that nervousness causes fear for me.<br><br>ARA641<br>Fear of consequences (reactions) resulting from nervousness	Two participants found the item unclear, with one questioning the justification of being scared when feeling nervous, and the other stating that nervousness causes fear. The researcher recommended a new translation, "عندما أتوتّر أشعر بالخوف" (When I get nervous, I feel scared), which the language consultant agreed with. However, the consultant noted that the tense needed to be changed to the past.
	**EDANX48 Many situations made me worry**	ARA647<br>Situations that occupy my mind.<br><br>ARA648<br>My mind was busy thinking about several situations.<br><br>ARA649<br>My mind is extremely worried about my kids.<br>ARA650<br>I was thinking of situations that happened to me.<br><br>ARA651<br>Negative situations occupied my mind however this was not specified in the item.	The participants' responses indicate a clear understanding of the item, with one participant mentioning that negative situations occupied their mind, although it was not specified in the item. The suggested translation change from " شغلت بالي مواقف عديدة" to "عدة مواقف أشغلت بالي" is accepted, as it clarifies the item and the change in word order does not pose any issues.
	**EDANX20 I was easily startled**	ARA652<br>Lack of focus<br><br>ARA653<br>Afraid	One participant chose "Sometimes" and mentioned being easily startled due to distractions. Two participants found the item unclear. The suggested translation "كنت أندهش بسهولة" means "I was getting puzzled/astonished" and was considered easier to understand. However, there was a suggestion to incorporate "أفزع" meaning "startled" instead of "puzzled/astonished." It was confirmed that "أفزع" was not used in any other items.

		ARA654<br>Unclear<br><br>ARA655<br>Afraid<br><br>ARA656<br>unclear	
